# Epigenetic regulation in cancer progression

**DOI:** 10.1186/2045-3701-4-45

**Published:** 2014-08-19

**Authors:** Eva Baxter, Karolina Windloch, Frank Gannon, Jason S Lee

**Affiliations:** 1 QIMR Berghofer Medical Research Institute, Control of Gene Expression Laboratory, Herston Rd, 4006 Herston, QLD, Australia

**Keywords:** Epigenetics, Hypoxia, Cancer, DNA methylation, Histone modifications, Acetylation, Demethylation, Transcription

## Abstract

Cancer is a disease arising from both genetic and epigenetic modifications of DNA that contribute to changes in gene expression in the cell. Genetic modifications include loss or amplification of DNA, loss of heterozygosity (LOH) as well as gene mutations. Epigenetic changes in cancer are generally thought to be brought about by alterations in DNA and histone modifications that lead to the silencing of tumour suppressor genes and the activation of oncogenic genes. Other consequences that result from epigenetic changes, such as inappropriate expression or repression of some genes in the wrong cellular context, can also result in the alteration of control and physiological systems such that a normal cell becomes tumorigenic. Excessive levels of the enzymes that act as epigenetic modifiers have been reported as markers of aggressive breast cancer and are associated with metastatic progression. It is likely that this is a common contributor to the recurrence and spread of the disease. The emphasis on genetic changes, for example in genome-wide association studies and increasingly in whole genome sequencing analyses of tumours, has resulted in the importance of epigenetic changes having less attention until recently. Epigenetic alterations at both the DNA and histone level are increasingly being recognised as playing a role in tumourigenesis. Recent studies have found that distinct subgroups of poor-prognosis tumours lack genetic alterations but are epigenetically deregulated, pointing to the important role that epigenetic modifications and/or their modifiers may play in cancer. In this review, we highlight the multitude of epigenetic changes that can occur and will discuss how deregulation of epigenetic modifiers contributes to cancer progression. We also discuss the off-target effects that epigenetic modifiers may have, notably the effects that histone modifiers have on non-histone proteins that can modulate protein expression and activity, as well as the role of hypoxia in epigenetic regulation.

## Introduction

Cancer initiation and progression have been recognised for many years to be secondary to the accumulation of genetic mutations which lead to changes in cellular function. While inherited or sporadic mutations may result in the activation of oncogenes or the inactivation of tumour suppressor genes, changes in modification of both DNA and histones (collectively the epigenome) can also contribute to the initiation and the progression of cancer. Although epigenetics is formally defined as a heritable change in gene expression or chromosomal stability by utilising DNA methylation, covalent modification of histones or non-coding RNAs without a change in DNA sequence, it is increasingly used to define long term changes that alter the physiology of a subset of cells in a tissue independent of a change in the DNA sequence. It should be noted that epigenetic marks are dynamic and can respond to changes in physiological conditions and hence, in addition to gene mutations, can be drivers of the development of the cancer. Global reprogramming of epigenetic marks, including alterations in DNA methylation and histone modifications, is known to occur in malignancy [1].

## Epigenetic regulation

Epigenetic modification of chromatin plays an important role in the regulation of gene expression. DNA is methylated post-synthetically on cytosine residues predominantly in the sequence CpG and *in vitro* methylated promoters are known to be generally inactive when transfected into eukaryotic cells [2]. DNA methylation is catalysed by a family of DNA methyltransferases (DNMTs): DNMT1 is the methyltransferase that maintains reciprocal methylation of the new DNA strand complementary to hemi-methylated DNA that is produced as a result of semi-conservative DNA replication. DNMT3a and DNMT3b are known as *de novo* methyltransferases, being able to methylate the completely unmethylated DNA duplex *in vivo*[3,4]. More recently it has been shown that 5-methylcytosine can be oxidised to 5-hydroxymethylcytosine by a family of Fe^2+^, 2-oxoglutarate dependent methylcytosine dioxygenases known as TET proteins [5], effectively resulting in the subsequent removal of the repressive methyl group by a mechanism that appears to include base excision repair processes. Other DNA modifications are also described such as methylation at sites other than CpG [6,7] and the generation of formyl and carboxyl derivatives of DNA [8].

**Figure 1:**
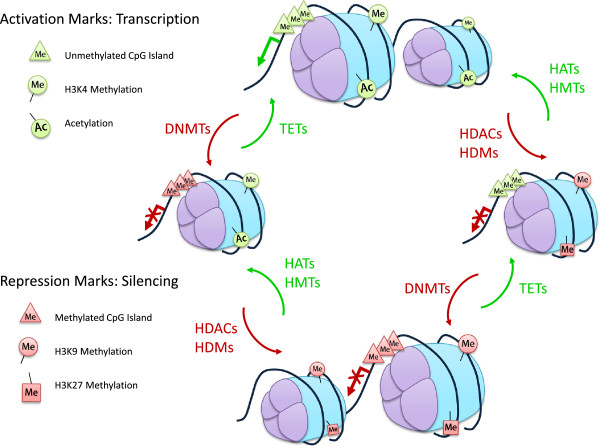
**Double Lock Principle.** A gene will be transcribed when it is in the open or “unlocked” state. The promoter region is demethylated, histones acetylated and H3K4me marked. If the gene is silenced, in a closed or “locked” state, DNA methyltransferases (DNMTs), histone deacetylases (HDACs), histone methyltransferases (HMTs) and histone demethyltransferases (HDMs) have modified the promoter region, removing the histone acetylation and modifying methylation accordingly. For the gene to be transcribed, the repression marks will need to be lifted to confer the open, “unlocked” state, by the TETs (removal of methylation on the promoter), histone acetyltransferases (HATs) and the HMTs/HDMs. If the DNA exists in any in-between state, with only partial silencing or activation marks, the gene remains repressed, hence the term “Double Lock”.

Earlier discussions that derived from those that studied transgenerational phenomena focused on the classical set of DNMTs. However, epigenetic modifications go beyond DNA methylation. The histone proteins in chromatin are also modified on their N-terminal residues and transcriptional states are frequently associated with particular histone modifications [9]. The number and complexity of the potential combinations of these has grown very rapidly in recent years [10] but a simplified generalisation could be that acetylation of histones H3 and H4 and methylation of the lysine-4 residue of histone H3 (H3K4) are associated with active genes. Inactive genes are frequently hypoacetylated and may also be methylated on the lysine-9 (H3K9) or lysine-27 (H3K27) residues of histone H3 (reviewed in [11]). Clearly there are possibilities for more complex situations when, for example, both H3K4 and H3K27 are methylated as occurs at bivalent domains in embryonic stem cells [12]. Although most studies tend to focus attention on either the DNA or histone modifications, it is clear that in order for a gene to be transcribed there is interplay between the methylated DNA and the modified histones. Both the DNA and the histones should be in an open or “unlocked” configuration, as shown in Figure 1, to be in a permissible state for transcription. If the epigenetic marks on the DNA or histones are in a closed or “locked” state, the gene of interest will not be transcribed. This is a concept that we term the “Double Lock Principle” as both the DNA methylation status and histone modifications are critical to the expression of a gene. In addition, the required transcriptional activator must be present and the necessity to have it and the “double lock” correctly aligned explains a lot of data where genes are not expressed despite what could be considered to be tolerant conditions.

Many enzymes have been identified that methylate, demethylate, acetylate, deacetylate, phosphorylate, ubiquitinate or sumoylate histones. There is redundancy and specificity in these enzymes that is required to deliver the full range of potential histone post-translational modifications. Additionally these enzymes may modify non-histone proteins such as Reptin and p53, contributing to their post-translational regulation (Table 1).

**Table 1:** Classification of epigenetic modifiers

**Class**	**Enzymes**		
**Histone Acetyltransferases (HATs)**	ELP3/KAT9	PCAF/KAT2B	MORF/MYST4/KAT6B
	GTF3C4	CBP/KAT3A	HBO1/MYST2/KAT7
	HAT3	p300/KAT3B	MOF/MYST1/KAT8
	HAT1/KAT1	Tip60/KAT5	KAT10
	GCN5/KAT2A	MOZ/MYST3/KAT6A	TFIIIC90/KAT12
**Histone Deacetylases (HDACs)**	HDAC1	HDAC7	SIRT2
	HDAC2	HDAC8	SIRT3
	HDAC3	HDAC9	SIRT4
	HDAC4	HDAC10	SIRT5
	HDAC5	HDAC11	SIRT6
	HDAC6	SIRT1	SIRT7
**Histone Methyltransferases (HMTs)**	ASH1	NSD1/KMT3B	SETD1A
	Clr4/KMT1	PRMT1	SETD8/Pr-SET7/KMT5A
	Dot1L/KMT4	PRMT3	SETDB1
	EZH2/KMT6	PRMT4/CARM1	SETDB2/KMT1F/CLL8
	G9a/EHMT2	PRMT5/JBP1	SMYD2/KMT3C
	GLP/EHMT1	PRMT6	SUV39H1
	KMT5B/KMT5C	Riz1/Riz2/KMT8	SUV39H2
	MLL1	NF20	SUV4-20H2/KMT5C
	MLL2	RNF40	TRX/ KMT2a
	MLL3	SET1A	HIF-1/ SET2/HYPB/KMT3A
	MLL4	SET1B	
	MLL5	SET7/9	
**Histone Demethylases (HDMs)**	ARID1A	JHDM1b/FBXL10/KDM2B	JMJD2D/KDM4D
	ARID5B	JHDM2A/KDM3A	JMJD3/KDM6B
	JARID1A/RBBP2/KDM5A	JHDM3A/JMJD2A/KDM4A	LSD1/KDM1
	JARID1B/PLU1/KDM5B	JMJD1A	LSD2
	JARID1C/SMCX/KDM5C	JMJD1B/KDM3B	PHF2
	JARID1D/SMCY/KDM5D	JMJD2A	PLU1
	JHD1/KDM2	JMJD2B/KDM4B	UTX/KDM6A
	JHDM1a/FBXL11/KDM2A	JMJD2C/GASC1/KDM4C	
**DNA Methyltransferases (DNMTs)**	DNMT1	DNMT3b	DNMT1o
	DNMT3a	DNMT3L	
**DNA Demethylases**	TET1	TET2	TET3

DNA methylation patterns and histone modifications have been found to be different when normal tissues and tumours derived from them are compared. All gene expression is ultimately controlled by their epigenetic status and it is not surprising therefore that epigenetic changes may play an important role in tumorigenesis. However, why these changes occur is unknown nor is it always clear if these changes are the causes of the tumour growth or if they are responding to altered environments (e.g. hypoxia). It is most likely that both sequences of events occur and irrespective of whether these are causes or consequences the epigenetic status is crucial to the cellular outcomes.

The enzymes mediating epigenetic modifications have been found to be mutated in cancers, which adds to an indirect manner in which tumours develop as the change in the modifier can affect the gene expression patterns. This suggests also that epigenetic modifiers may act as novel targets for therapy. Mutations of DNMT3a have been observed in 22% of cases of acute myeloid leukaemia (AML) where they are associated with a poor outcome [13]. Similarly, the methylcytosine dioxygenase TET2 is mutated in ~15% of myeloid cancers [14]. *Tet2*-deficiency in mutant mice causes myeloproliferation, suggesting a role in stem cell function [15]. The H3K27 demethylase UTX is mutated in multiple human cancers, the highest frequency (~10%) being in multiple myeloma [16]. The discovery of mutations in genes that modify chromatin suggests that the disruption of epigenetic control has a very significant role in the promotion of cancers. There are also secondary roles where specific proteins bind to correctly modified histones. Alteration in their structure can also drive the development of tumours. For example ASXL1 (additional sex comb-like 1) is a member of the Polycomb group of proteins that bind modified histones and is mutated in 11% of myelodysplastic syndromes and 43% of chronic myelomonocytic leukaemias [16,17].

## DNA methylation

The status of DNA methylation is crucial as one part of the “double lock” of gene expression. As a generalisation, promoters with methylated DNA tend not to be expressed. Clusters of CpGs (the predominant target for DNA methylation) are known as CpG islands and are located at the 5′ ends of many human genes. In tissues, most CpG islands are unmethylated, even when the associated genes are not expressed [18]. However in cancer, DNA hypermethylation occurs at many CpG islands, as well as global DNA hypomethylation (discussed in DNA demethylation section). Promoter methylation is almost always associated with gene-silencing, raising the possibility that aberrant methylation might cause silencing and be part of the transforming process. A potential role in tumorigenesis with a strong mechanistic pathway is suggested when methylation is shown to occur at known tumour suppressor genes. DNA hypermethylation of the cell cycle control gene RB (retinoblastoma) was one of the first epigenetic lesions to be implicated in carcinogenesis. Aberrant methylation occurs in approximately 10% of cases of sporadic unilateral retinoblastoma [19] and is associated with the loss of RB expression [20]. The case of DNA methylation in RB remains one of the strongest arguments in favour of a causal role for aberrant methylation in carcinogenesis as the RB gene is usually active in the precursor cells of tumours and promoter methylation appears to have the same effect as genetic mutation of the gene [21]. Another tumour type in which this occurs is microsatellite unstable colon cancer. Inherited forms of the disease are frequently caused by germline mutation of the DNA mismatch repair (MMR) protein MLH1 [22]. However, approximately 15% of cases of sporadic colon cancer lack MMR gene mutations yet still exhibit microsatellite instability [23]. These cases have methylated MLH1 promoters and lack expression of the gene [24]. In cell lines showing this abnormality, the MLH1 repression is reported to be reversed by treatment with the demethylating agent 5-aza-2′-deoxycytidine [25]. Another in a growing number of examples is the aberrant methylation of the p16^INK4a^/CDKN2A promoter which has been shown to be present in both human squamous cell carcinomas and their precursor lesions [26], indicating that it occurs in the early stages of neoplastic transformation. Similarly, methylation of GSTP1 (π-class glutathione S-transferase) is an early event in prostate carcinogenesis as it is also found in premalignant lesions [27]. In colorectal carcinogenesis, hypermethylation of a region of chromosome 17p corresponding to the location of the tumour suppressor p53 has been demonstrated to precede its allelic loss, suggesting that methylation may non-randomly mark chromosome regions that are altered during the development of specific tumours [28]. Because of these examples, it has been assumed that aberrant methylation plays a role in malignant transformation [1], particularly when methylation has been demonstrated to occur early in the tumorigenic process. The methylation-induced silencing of tumour suppressor genes may provide cells with a selective advantage over others, either by causing their increased proliferation or resistance to apoptosis. The clonal expansion of these premalignant cells could result in the hyperproliferative phenotype that is characteristic of the early stages of tumorigenesis [29]. Genes such as RB, MLH1 and VHL are methylated in the tumour types in which they are also commonly mutated, suggesting that CpG island hypermethylation may be selected for during tumorigenesis [30].

DNA hypermethylation has been used to subdivide tumour types and distinguish them from non-malignant tissue [31]. Tumour subgroups with high levels of DNA methylation have been designated as having a CpG island methylator phenotype (CIMP) and are predominantly associated with worse prognosis. CIMP was first identified in colorectal tumours where they encompass the majority of sporadic colorectal cancers with MMR-deficiency and MLH1 hypermethylation [32] and are specifically associated with the BRAF^V600E^ mutation [33]. CIMP has subsequently been found to define a subset of glioblastomas [34], acute myeloid leukaemias [35], gastric cancers [36] and ependymomas [37]. CIMP tumours may thus represent distinct subgroups of tumours which otherwise have few genetic alterations, suggesting that drugs targeting the epigenetic machinery may offer novel approaches for therapy.

## DNA demethylation

DNA demethylation has also been postulated to contribute to cancer development as despite evidence for regional hypermethylation, global levels of 5-methylcytosine have actually been found to be 5-10% less in tumours compared to normal cells [38,39]. The methylation changes have been suggested to occur specifically between the stages of hyperplasia and benign neoplasia as DNA was found to be significantly hypomethylated in both benign polyps and malignant tissues when compared to normal tissue [40]. Methylation patterns were therefore altered before the lesions became malignant, suggesting that they could be a key event in tumour evolution. The cause of global hypomethylation in cancer is unknown but the outcome, in due course, may be that oncogene expression is increased or other genes important for growth control are deregulated.

Several mechanisms have been proposed for the demethylation of DNA; passive demethylation may occur due to the inability of the maintenance methyltransferase to complete the methylation step that would normally be guided by hemi-methylated DNA post-replication. This is thought to be the case for the maternal pronucleus which undergoes passive demethylation during pre-implantation development, most likely due to sequestration of the oocyte-specific form of DNMT1 (DNMT1o) in the cytoplasm throughout most of cleavage [41]. Conversely, rapid demethylation of the paternal pronucleus appears to be due to the oxidation of 5-methylcytosine to 5-hydroxymethylcytosine by TET3 [42]. There is evidence that the maintenance methyltransferase DNMT1 does not restore methylation to cytosines in the newly synthesised daughter strand if the diagonally opposite cytosine on the parent strand is hydroxymethylated [43], resulting in replication-dependent passive dilution of 5-methylcytosine. Active DNA demethylation in cultured human cells and the adult mouse brain has been demonstrated to involve TET1-catalysed hydroxymethylation, followed by AID/APOBEC-mediated deamination of 5-hydroxymethylcytosine, with the resulting base mismatch being removed by the base excision repair pathway [44]. TET proteins are also able to further oxidise 5-hydroxymethylcytosine to 5-formylcytosine and 5-carboxylcytosine which can be excised by TDG (thymine DNA glycosylase) and repaired by the base excision repair pathway [45,46]. In a study that examined the methylation status of a number of genes when the cells were released from a synchronising block, DNA methylation and demethylation have been shown to cycle approximately every hour ([47,48]. This was a surprise discovery that permits different possibilities including a dynamic, replication-independent response to changes in physiological conditions such as hypoxia. One mechanism that has been proposed is that TDG and components of the base excision repair pathway were recruited to the promoter at the beginning of each transcriptionally productive cycle and a reduction in TDG expression impaired demethylation and reduced transcriptional activity [48].

Contrary to expectations, loss-of-function of the methylcytosine dioxygenase TET2 is predominantly associated with loss of DNA methylation [49]. TET2 is mutated in ~15% of myeloid cancers [14], resulting in impaired hydroxylation [49]. TET2 function is also inhibited by the oncometabolite 2-hydroxyglutarate generated by mutant IDH1 in acute myeloid leukaemias [35]. Downregulation of TET expression has been reported in breast and liver cancers, with reduced levels of 5-hydroxymethylcytosine [50]. DNA methylation patterns may thus be modified by altered expression or activity of epigenetic regulators such as TET.

## Histone modifications

Chromatin remodelling is by the so called “histone-code” involving various covalent modifications of the histones such as acetylation, phosphorylation and methylation which have been subject to many studies and their importance is now well accepted [51]. However, the transcriptional state can also be regulated by many chromatin-associated protein complexes that are either involved in enhancing or fine-tuning of the promoter activity and some of these respond to the altered contexts that arise from the histone and DNA modifications. The histone methylation balance on specific residues in particular is crucial for maintaining genome integrity, gene expression and evasion of cancer [10,52,53].

Misregulation of the histone methyltransferases (HMTs) and the histone demethylases (HDMs) has been associated with a variety of cancer types including breast, prostate, lung and brain [54-58]. Specifically, the HMTs and the HDMs play important roles in multiple tissues regulating the methylation status of four lysine residues K4, K9, K27 and K36 on histone H3. Similar to DNA methylation patterns, histone modification patterns have also been used to predict prognosis in multiple cancers. Reduced levels of H3K9ac, H3K9me3 and H4K16ac correlated with recurrence of non-small cell lung cancer [59]. In prostate cancer, lower levels of H3K4me2 and H3K18ac were associated with poor prognosis [60]. Loss of H3K9me3 has been found in core promoter regions of genes in patients with acute myeloid leukaemia. Global H3K9me3 patterns were additionally able to independently predict patient prognosis in acute myeloid leukaemia [61]. These cancers have amplifications, deletions and somatic mutations which all lead to changes in the enzymatic activities of the HMTs and the HDMs. For example, the repressive histone mark trimethylated H3K27 (H3K27me3) is mediated by the catalytic SET domain of EZH2 (enhancer of zeste homologue 2), a protein that forms part of PRC2 (Polycomb repressive complex 2). EZH2 has been reported to be up-regulated in metastatic prostate cancer relative to localised disease or benign prostatic hypertrophy, suggesting a potential involvement in prostate cancer progression [57], and its over-expression also correlates with breast cancer aggressiveness and poor prognosis [56]. The H3K9 methyltransferase G9a reportedly promotes lung cancer invasion and metastasis by silencing Ep-CAM [55]. It is also known that hypoxia in tumours can influence methylation of the histone H3K9 as well as the chromatin remodelling factors by increasing G9a protein stability [62-64]. It should be noted that here, as was the case in consideration of the role of DNA methylation, it is the switching off of gene expression that drives tumour progression. Even though there is an equal possibility for genes that are deleterious to be switched on through changes in the enzymes that alter the epigenome, it would seem that the switching off of genes is the crucial trigger for the progression of tumours through altering the inherent stable balance in cells.

In order to maintain methylation balance, several “histone” demethylases exist which demethylate specific residues, i.e. the reverse of the action of the methyltransferases on different histone residues. There are two classes of HDM families identified which use distinct biochemical reactions to achieve demethylation. Lysine-specific demethylase 1 (LSD1) was the first enzyme identified to demethylate H3K4me1 and H3K4me2, and later found to also demethylate H3K9me1 and H3K9me2 [65,66]. LSD1 is known to utilise flavin adenine dinucleotide (FAD)-dependent amine oxidation reaction for demethylating its substrates and appears to be a very promiscuous protein, having the ability to interact with many proteins and to be involved in multiple biological functions. It should be noted that a potential linkage between metabolic state and gene expression arises from the use of this co-factor and this may be crucial to ensure that it does not destabilise the epigenome. The second class of demethylases includes several proteins that possess a catalytic JMJC domain. These enzymes demethylate histone residues through a dioxygenase reaction which depend on Fe (II) and α-ketoglutarate as cofactors. Again it is interesting to note the crucial role of a metabolite which suggests that the integration of diverse cellular processes and the environment in which the cell resides is decisive on defining the pattern of genes that will be expressed or repressed. It is self evident that some such process is a necessary integrator of cell physiology. Unlike LSD1, JMJC domain-containing demethylases such as JHDM3A have the ability to demethylate trimethylated histone H3K9 and H3K27 residues [67,68]. More recently, deregulation and mutations that affect the enzymatic activity have been found for the HDMs. The H3K27 demethylase JMJD3 is found to be down-regulated in liver and lung cancers [58] while inactivating somatic mutations in the UTX gene are frequently found in multiple tumour types [16]. Knock-out mouse models of some of these HDMs have been generated and result in distinct phenotypes [68,69] including many that are lethal, indicating that proper expression of HDMs is crucial for development [69,70].

## Non-histone methylation

**Figure 2:**
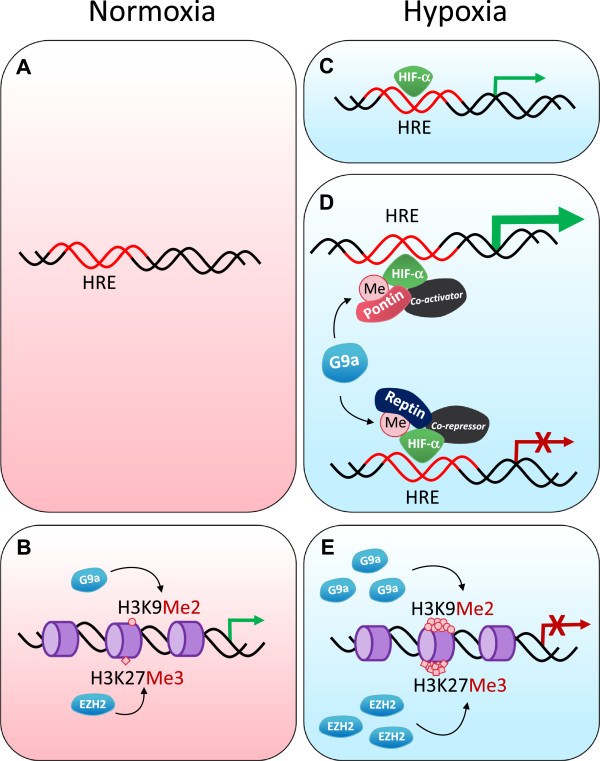
**Transcriptional control in normoxia and hypoxia. (A)** In normoxia, proteasomal degradation of HIFs prevents HIF-α binding to a hypoxia response element (HRE) and transcriptional activation does not occur. **(B)** The expression of other genes can be regulated by methylation at histones H3K9 and H3K27 by G9a and EZH2 respectively to maintain homeostasis. **(C-E)** In hypoxia, gene expression is regulated at multiple layers; **(C)** HIF-α is stabilised in hypoxia and is able to bind to HREs and activate transcription. **(D)** The transcriptional activity of HIF-α can be modulated by co-regulators; G9a methylates chromatin remodelling complex proteins such as Reptin and Pontin in hypoxia. Methylated Reptin negatively regulates transcriptional activation by HIF-α at a subset of HIF-α target genes by recruiting a transcriptional co-repressor. Conversely, Pontin methylation potentiates HIF-α-mediated transcription at another distinct subset of HIF-α target promoters by enhancing the recruitment of a transcriptional co-activator. **(E)** The expression of histone methyltransferases such as G9a and EZH2 is elevated in hypoxia which leads to silencing of tumour suppressors through the hypermethylation of histones H3K9 and H3K27.

Although their name arises from the first substrate that was associated with them, several proteins other than histones have been identified to be methylated by the HMTs and also demethylated by the HDMs [71-73]. The tumour suppressor protein p53 was one of the first non-histone substrates identified to be methylated by several HMTs including Set9, smyd2 and G9a [71,72,74] and also demethylated by LSD1 [66,73]. Depending on which lysine residue is methylated, the transcriptional activity of p53 is specifically regulated. Methylation of non-histone proteins by HMTs has been shown to result in a range of outcomes ranging from functional activation [64,75] to repression [76] or degradation [77]. Hypoxia induces methylation of the chromatin remodelling protein Pontin by stabilising G9a. Methylated Pontin interacts with p300 histone acetyltransferase and HIF-α to hyperactivate a subset of HIF-α target genes [64] (Figure 2). G9a also increases methylation of another chromatin remodelling protein Reptin in a hypoxia-dependent manner. Unlike Pontin methylation, Reptin methylation results in negative regulation of a distinct subset of HIF-α target genes [63]. Two non-histone substrates of EZH2 have been reported recently both of which represses its transcriptional activity. GATA4 is methylated by EZH2 which reduces its interaction with its coactivator p300 [76]. Our group has shown that methylation of the nuclear receptor RORα by EZH2 results in increased polyubiquitination and proteasomal degradation leading to decreased transcriptional activity [77]. In turn this causes the loss of tumour suppressor activity of RORα, which ultimately leads to the development of more aggressive tumours.

It is not only the histone methyltransferases that interact with various non-histone proteins, we have also found that one of the HDMs (JMJD1A) interacts with several proteins, possibly targeting them for demethylation. Therefore, the net status of protein methylation appears to have a broad range of biological functions. Although the dynamic nature of this non-histone methylation appears to be important just as it is the case for histones, demethylation of these proteins has not been studied extensively.

## Tumour hypoxia and regulation of gene expression

Tumour hypoxia is an example of how epigenetic reprogramming occurs in cancer progression. In solid tumours, hypoxia occurs as a result of the limitation of oxygen diffusion in avascular primary tumours or their metastases. Persistent hypoxia significantly reduces the efficacy of radiation and chemotherapy and leads to poor outcomes. This is mainly due to increases in pro-survival genes that suppress apoptosis such as c-myc, AMPK, GLUT1 and BNIP3 [78-81] and enhance tumour angiogenesis, EMT (epithelial-to-mesenchymal transition), invasiveness and metastasis [82,83].

Much of tumour hypoxia research has been centred on examining the transcriptional targets of HIFs (hypoxia-inducible factors). HIF-α is a heterodimeric transcription factor that is comprised of an oxygen-regulated α subunit (HIF-1α or HIF-2α) and a constitutively expressed β subunit (HIF-1β) [84,85]. HIF-α is an oxygen-responsive transcription factor that mediates adaptation to hypoxia. Under low oxygen concentrations, HIF-α is stabilised and translocates to the nucleus, leading to specific target gene expression through binding of HIF-1β to a hypoxia response element (HRE). HIF-α regulates hundreds of genes involved in many biological processes including tumour angiogenesis, glycolysis, invasion, metabolism and survival and hence dramatically changes the functioning of cells that reside in these conditions.

Hypoxia not only activates gene expression, but is also involved in gene repression. While some of these genes are known to be transcriptionally downregulated by the recruitment of specific repressors such as DEC1 and Snail [54,86], the contribution of hypoxia-driven epigenetic regulation to gene silencing remains unclear. It has been shown that the expression of G9a and EZH2 are elevated in hypoxic conditions, leading to global hypermethylation of H3K9 and H3K27 respectively. These repressive modifications were increased by hypoxia in the promoter regions of tumour suppressor genes such as RUNX3 and MLH1 which correlated with their silencing, potentially promoting tumour progression [62,87]. We have found that the activity of G9a is deregulated in a tumour setting; methylation of the non-histone proteins Reptin or Pontin in hypoxic conditions negatively or positively regulates the transcription of a particular set of genes involved in tumour metastasis [63,64] (Figure 2).

## Conclusions

There has been significant attention in the literature to the accumulated changes in DNA sequences that ultimately give rise to tumours being formed. This has resulted in a rather simple model of tumourgenesis based on accumulated random mutations. In this article we focus on the role of the epigenome as an alternative mode of acquiring dysfunctional cells that result in cancers. Having indicated the necessity to have both the DNA and histone modifications correctly aligned such that the expression of a gene occurs, we point to the plethora of modifying enzymes that can have roles to play. These enzymes with their ability to switch on or off genes have every possibility to change a benign cell into one that is cancerous. Indeed their normal function is to ensure that the correct genes are expressed and that the level of this expression and its timing are all coordinated such that a physiologically normal cell exists. It is clear that any perturbation from this state can have the effect of either making the cell non-viable or to grow to an excessive level and hence become a tumour. A systems-based approach is hence needed to fully integrate all of the available information. What is clear is that both DNA and histone hypermethylation and hypomethylation (and in the case of histones the acetylation state) are associated with malignancy, indicating that balanced epigenetic control is required. Targeting epigenetic modifiers presents novel strategies for cancer therapy in both treating disease and delaying or even preventing resistance to other therapies such as aromatase inhibitors. A recent report found that extended use of aromatase inhibitors resulted in the recruitment of EZH2 and hence increased H3K27me3 of the homeobox gene HOXC10 in breast cancer cells, ultimately leading to HOXC10 methylation and silencing and resistance to aromatase inhibitors [88]. The DNA demethylating agents 5-azacytidine and 5-aza-2′-deoxycytidine (decitabine) and HDAC inhibitors SAHA (vorinostat) and romidepsin have been approved for clinical use with the aim of reversing gene silencing mediated by the DNA methyltransferases or histone deacetylases. These growing numbers of examples point to great complexity and crossover mediated by epigenetic changes between the different inhibitors in clinical use. Given the close interplay between DNA methylation and histone modifications, dual therapy targeting both types of epigenetic modifications may be required. Selected novel drugs targeting components of the epigenetic machinery are currently in pre-clinical or clinical development. Care should, however, be taken in inhibiting epigenetic modifiers due to their off-target effects as illustrated by the non-histone targets for histone modifying enzymes.

## Competing interests

The authors declare no financial or non-financial competing interests.

## Authors’ contributions

JSL, EB and FG wrote the manuscript. KW generated the figures. All authors read and approve the final manuscript.
